# Subclinical doses of dietary fumonisins and deoxynivalenol cause cecal microbiota dysbiosis in broiler chickens challenged with *Clostridium perfringens*

**DOI:** 10.3389/fmicb.2023.1106604

**Published:** 2023-04-03

**Authors:** Revathi Shanmugasundaram, Jeferson Lourenco, Walid Al Hakeem, Madison M. Dycus, Todd J. Applegate

**Affiliations:** ^1^Toxicology and Mycotoxin Research Unit, U.S. National Poultry Research Center, Agricultural Research Service, U.S. Department of Agriculture, Athens, GA, United States; ^2^Department of Animal and Dairy Science, University of Georgia, Athens, GA, United States; ^3^Department of Poultry Science, University of Georgia, Athens, GA, United States

**Keywords:** fumonisins (FB1, FB2), deoxynivalenol (DON), cecal microbiome, *Clostridium perfringens*, necrotic enteritis, poultry

## Abstract

Fusarium toxins are one of the most common contaminants in poultry diets. The co-occurrence of fumonisins (FUM) and deoxynivalenol (DON), even at a subclinical dose, negatively affects the growth performance, intestinal integrity and induce subclinical necrotic enteritis in broiler chickens. Loss of gut integrity can be expected to alter the intestinal microbiota’s composition. The objective of this study was to identify the effects of combined FUM and DON on the cecal microbiome profile and predicted metabolic functions and a short chain fatty acid profile in broilers challenged with *Clostridium perfringens*. A total of 240 1 day-old chicks were randomly assigned to two treatments: a control diet and the control diet with 3 mg/kg FUM + 4 mg/kg DON each with eight replications. All the birds were received cocci vaccine at d0. All birds in both treatment groups were challenged with *C. perfringens* 1 × 10^8^ CFU *via* feed on d 19 and 20 to achieve 5% mortality. On d 35, the FUM and DON contaminated diet numerically (*P* = 0.06) decreased the body weight gain (BWG) by 84 g compared to the control group. The bacterial compositions of the cecal contents were analyzed by sequencing the V3–V4 region of the 16S rRNA gene. Overall, microbial richness and diversity increased (*P* < 0.02) during the studied period (d 21–35). Cecal contents of birds in the FUM + DON group had greater (*P* < 0.05) microbial evenness and diversity (Shannon index) compared to the control group. FUM + DON exposure decreased (*P* = 0.001) the relative abundance of *Proteobacteria* in the cecal content, compared to the control group. The combined FUM + DON significantly increased the relative abundance of the *Defluviitaleaceae* and *Lachnospiraceae* families (*P* < 0.05) but decreased the abundances of the *Moraxellaceae* and *Streptococcaceae* (*P* < 0.05) compared to the control group birds. At the genus level, FUM + DON exposure decreased (*P* < 0.05) *Acinetobacter* and *Pseudomonas* abundance and had a tendency (*P* = 0.08) to decrease *Thermincola* abundance compared to the control group. In the ileum, no NE-specific microscopic abnormalities were found; however, the tip of the ileal villi were compromised. The present findings showed that dietary FUM and DON contamination, even at subclinical levels, altered cecal microbial composition, dysregulated intestinal functions, and impaired the gut immune response, potentially predisposing the birds to necrotic enteritis.

## Introduction

The chicken’s gut microbial community is complex, and the interactions between gut microbiota significantly affect the physiological, immunological, nutritional (Zhao et al., 2013), and health status (Kumar et al., 2018) of the host. The gut microbial community directs the development of the host immune system by interacting with the host innate immune system and plays a role in the development of T-cell or B-cell repertoires (Pan and Yu, 2014; Kogut and Arsenault, 2016). When compared to mammals, the chicken gastrointestinal tract is shorter, with an average feed passage time of less than 3.5 h (Hughes, 2008). However, slow feed retention times in ceca provide an ideal site for some microbial taxa, such as *Bacteroidaceae*, *Ruminococcaceae*, *Lachnospiraceae*, and *Clostridiaceae*, to thrive in the cecal microenvironment (Oakley et al., 2014).

Mycotoxins are ubiquitously present in both pre- and post-harvest feed and food commodities. The annual estimated loss due to all mycotoxins in agriculture is approximately $1.4 billion (Vardon et al., 2003). Among *Fusarium* mycotoxins, fumonisins (FUM) and deoxynivalenol (DON) are commonly present in corn and other poultry feed ingredients (Awad et al., 2013). The epithelial cells in the chicken gastrointestinal tract are the very first target cells for the mycotoxins (Grenier and Applegate, 2013). Mycotoxins cause intestinal damage, activate gut inflammation, and eventually disturb gut microbiota homeostasis (Elmassry et al., 2022). Gut microbiota are considered key players and play a pivotal role in the detoxification of orally ingested mycotoxins (Grenier and Applegate, 2013; Liew et al., 2018).

Mycotoxins reduce chicken growth performance by altering gut bacterial composition and interactions between gut microbes and the host (Robert et al., 2017). Chronic subclinical doses of either FUM or DON alone affect the gut microbiome diversity (Antonissen et al., 2015; Lucke et al., 2018). DON contamination at 10 mg/kg diet decreases cecal species richness and evenness in broiler chickens. Similarly, 12 mg/kg of total FUM contamination altered the composition of the bacterial community and decreased the microbial diversity index in pigs (Burel et al., 2013; Mateos et al., 2018).

The current FDA recommendation is that the maximum concentrations of DON and FUM should be 5 and 50 mg/kg in the finished diet of broiler chickens, respectively. However, these concentrations are for individual mycotoxins, and according to a 2021 mycotoxin survey, 58% of the corn samples tested were positive for more than one mycotoxin (Biomin, 2021). Further, *Fusarium* species such as *F. verticillioides* and *F. graminearum* produce multiple mycotoxins and secondary metabolites, including trichothecenes. As a result, the co-occurrence of multiple mycotoxins in poultry finished diets cause a synergistic detrimental effect on poultry health and adversely affects production performance, immunity, and health status.

Necrotic enteritis is a disease with multiple etiologies. Uncontrolled proliferation of *Clostridium perfringens* in the gut following coccidial pathogen-induced gut damage has been recognized as a major etiological factor that induces necrotic enteritis in broilers (Cooper and Songer, 2009). We earlier reported that continuous exposure to 3 mg/kg of FUM and 4 mg/kg of DON, at doses below FDA tolerance concentrations, induces subclinical necrotic enteritis in chickens even in the absence of coccidial-induced gut damage but in the presence of *C. perfringens* inoculation. FUM and DON-induced losses in gut integrity and the presence of *C. perfringens* resulted in subclinical necrotic enteritis (Shanmugasundaram et al., 2022). The objective of this study was to identify if subclinical doses of combined FUM and DON alter cecal microbial composition or their microbial functions and the SCFA profile in broiler chickens challenged with *C. perfringens*, and thereby explain the occurrence of subclinical necrotic enteritis in birds exposed to FUM and DON.

## Materials and methods

### Diet formulation

Two strains of *Fusarium*, *F. graminearum* strain PH-1 and *F. verticillioides* strain M3125, were cultured for DON and FB production, respectively, as mentioned previously (Shanmugasundaram et al., 2022). A non-medicated corn–soybean meal-based mash diet was used as a basal diet (Supplementary Table 1). The feeding experiment was divided into two phases: (1) the starter diet (d0–18) and (2) the finisher diet (d 19–35). The final diets were analyzed to determine the actual content of FB, DON, and other major mycotoxins by LC-MS-MS in Romer Labs (Union, MO, United States). The mycotoxin content of the experimental diets is provided in Supplementary Table 2.

### Birds and housing

A total of 240 1 day-old Ross x Ross 708 strain broiler chicks (Aviagen, Blairsville, GA, USA) were weighed individually and randomly distributed to either one of the two treatments: control or FUM (3 mg/kg diet) + DON (4 mg/kg diet) contaminated diet. Each treatment was replicated in eight pens with 15 birds per pen, and the study was terminated on d 35. All animal protocols were approved by the Institutional Animal Care and Use Committee at the Southern Poultry Research Group, Athens, GA, and the birds were raised under the supervision of a licensed poultry veterinarian. The animal care practices and use procedures were followed under the *Guide for the Care and Use of Agricultural Animals in Research and Teaching* (McGlone, 2020). Day-old broiler chicks were raised in 1.5 × 1.5 m floor pens on new shavings or litter, as is standard industry practice in North America, and were kept at room temperature. The floor pens were equipped with nipple-type waterers and thermostatically controlled heaters. Chicks had *ad libitum* access to feed and water throughout the experimental period. The mortality of birds was recorded daily. All birds were euthanized by cervical dislocation, which was approved by the American Veterinary Medical Association.

### Production performances and *C. Perfringens* challenge

At 19 and 20 days, birds in both treatment groups were challenged with 1 × 10^8^ CFU of *C. perfringens* (strain #6) *via* feed, as described earlier (Hofacre et al., 1998). Prior to the *C. perfringens* challenge, feed and water were withdrawn from the birds for 4 and 2 h, respectively. On d 21, the mortality was reached 5% in the FUM + DON group; hence, both treatment groups had not received *C. perfringens* on d 21. Bodyweight and feed intake were measured at 7, 14, 20, 27, and 35 days of age. Average feed intake and body weight gain (BWG) were corrected for mortality when calculating the feed conversion ratio (FCR) for each pen.

### Total IgA quantification and *C. perfringens* specific IgA quantification in bile by ELISA

At 21, 28, and 35 days, bile was collected from one bird in each of the eight floor pens per treatment group (*n* = 8) and stored at –20^°^C until further use. The bile samples were collected from gallbladder using a 22 G needle. The bile total IgA quantification was measured by indirect ELISA using a chicken IgA ELISA kit following the manufacture’s instructions (Bethyl Laboratories Inc., Montgomery, TX, USA, Catalog # E33-103). Bile samples were also analyzed for anti-Clostridium IgA content using an ELISA as described previously (Shanmugasundaram et al., 2020). The primary and secondary antibody concentrations were confirmed by using checkerboard titrations with dilutions of bile, coating antigens, and secondary antibodies. The *C. perfringens* antigen for coating was prepared by six consecutive freeze-thaw cycles of pure culture of *C. perfringens* followed by mechanical lysing. The pure culture was lysed three times by glass beads of sizes 425–600 μm (Sigma-Aldrich, St. Louis, MO, USA) in a TissueLyser LT (Qiagen, Germany) for 5 min at 50 Hz. The lysed cells were centrifuged at 10,000 × g for 10 min, and the resultant supernatant was collected and stored at –70^°^C until use. Flat-bottomed 96-well microtitration plates (Microlon 600 High Binding, Greiner, NC, USA) were coated with 100 μL of 10 μg/mL of the antigen diluted in 0.1 M carbonate buffer and incubated overnight at 4^°^C. The plates were washed three times with PBS-Tween 20. To prevent non-specific binding, wells were blocked with 100 μL of SuperBlock™ (PBS) Blocking Buffer (Thermofisher Scientific, Waltham, MA, USA) and incubated for 30 min at 37^°^C. For IgA analysis, 100 μL of 1:1600 dilution of the bile in SuperBlock™ (PBS) Blocking Buffer was added to the plates in duplicates and incubated for 1 h at room temperature. After washing, 100 μL of 1:100,000 dilutions of horseradish peroxidase-labeled anti-chicken IgA (Novus Biologicals, Littleton, CO, USA) in SuperBlock™ (PBS) Blocking Buffer was added to each well and incubated for 1 h at room temperature. The plates were washed with PBS-Tween 20, and the substrate 3,3,5,5-tetramethylbenzidine (TMB) solution (eBioscience, San Diego, CA, USA) was added to the wells (100 μL/well). The reaction was stopped after 10 min using 1N HCl (100 μL/well), and the plate was read at 450 nm optical density using a microplate ELISA reader. IgA values were reported as the mean optical density.

### Ileum and jejunum histology

On d 21, 28, and 35, approximately 4 cm of jejunal and ileal samples were cut proximally and distally to the Meckel’s diverticulum. Samples were collected from 1 bird/pen (*n* = 8) from each replication post-Clostridium challenge and stored in buffered formalin. The jejunal and ileal samples were processed using a tissue processor (Sakura Finetek USA, Inc., Torrance, CA, USA) and embedded in paraffin. Paraffin blocks were cut into 5 μm cross-sections and mounted on superfrost slides (Thermo Fisher Scientific, Waltham, MA, USA) and stained with hematoxylin and eosin. Cross-sections were viewed using the cellSens Imaging software (Olympus America, Central Valley, PA, USA) to analyze the morphological differences. The NE microlesion score used in this study has previously been reported by Bortoluzzi et al. (2019). The following NE scores were used in this study: (0)- no necrotic lesions, intact intestinal epithelial cells attached to the lamina propria; (1)- mild single cell enterocyte degeneration/necrosis detachment from the lamina propria; (2)- mild necrosis at villi tips; (3)- moderate necrosis affecting half of the villi; (4)- severe necrosis affecting the entire villi and/or submucosa.

### Cecal DNA isolation and 16S rRNA gene amplification

At days 21, 28, and 35, ceca were removed from each of the eight floor pens per treatment group (*n* = 8), and cecal content was squeezed into 15 ml tubes that were immediately placed on ice before being stored at –20^°^C until further use. Bacterial genomic DNA was extracted using a combination of mechanical and enzymatic methods. Briefly, approximately 100 mg of cecal content samples were homogenized by using Lysing Matrix E Tube (MP Biomedicals, Solon, OH, USA) with the FastPrep homogenizer (MP Biomedicals, LLC, Irvine, CA, USA) at 6.0 m/s for 40 s, followed by genomic DNA extraction using the QIAamp PowerFecal Pro DNA Kit (Qiagen, Carlsbad, CA, USA) according to the manufacturer’s recommendations. The purified DNA was eluted in a total volume of 75 μL and the DNA concentrations were determined by a Synergy HT Microplate Reader using a Take3 plate (BioTek Instruments, Inc., Winooski, VT, USA). Bacterial genomic DNA samples were sent to LC Sciences (Texas, USA) for sequencing on an Illumina platform. The V3–V4 region of the bacterial 16S rDNA gene was amplified using the S-D-Bact-0341-b-S-17 (5′-CCTACGGGNGGCWGCAG-3′) forward and S-D-Bact-0785-a-A-21 (5′-GACTACHVGGGTATCTAATCC-3′) primer pairs (Klindworth et al., 2013). Each PCR reaction contained the DNA template (12.5 ng), 5 μL forward primer (1 μM), 5 μL reverse primer (1 μM), 12.5 μL 2 × Kapa HiFi Hotstart ready mix (Roche Sequencing and Life Science, Wilmington, MA, USA), and water to a final volume of 25 μL. The DNA was subjected to initial denaturation at 95^°^C for 3 min. Amplification was then achieved by 25 cycles of denaturation at 95^°^C for 30 s, annealing at 55^°^C for 30 s, and extension at 72^°^C for 30 s. The final extension was at 72^°^C for 5 min. PCR products were cleaned using AMPure XP magnetic beads (Beckman Coulter Life Sciences, Indianapolis, IN, USA) and 80% ethanol. The PCR products were submitted to another round of PCR to incorporate indexes (Illumina Nextera XT indexing primers, Illumina, Inc., San Diego, CA, USA) into the samples. Each PCR reaction contained 5 μL of each index primer, 25 μL 2 × Kapa HiFi hot start ready-mix (Roche Sequencing and Life Science, Wilmington, MA, USA), and 10 μL water. PCR cycling conditions were as previously described except for the number of amplification cycles, which was set to eight. PCR products were cleaned using AMPure XP beads and 80% ethanol, pooled, and paired ends were sequenced at a read length of 250 nucleotides on a NovaSeq platform (Illumina, Inc., San Diego, CA, USA).

### Short chain fatty acid analyses

At d 21, 28, and 35, chicken cecal content samples were analyzed for acetate, propionate, butyrate, valerate, isovalerate, and isobutyrate (SCFA) by using gas chromatography as described earlier (Lourenco et al., 2020). Briefly, the cecal content samples were mixed with three parts water and stored overnight. The samples were thawed and centrifuged at 10,000 × g for 10 min. A total of 1 mL of the supernatant was transferred into a new centrifuge tube, mixed with metaphosphoric acid solution (25% wt/vol) and the samples were frozen overnight. The samples were then thawed and centrifuged at 10,000 × g for 10 min. A total of 1 mL of supernatant was mixed with an internal standard (2-ethylbutyric acid), and ethyl acetate in a 2:1 ratio. After homogenization, samples were allowed to settle for 5 min, and then 0.5 mL of the separated ethyl acetate fraction was analyzed by gas chromatography (Shimadzu GC-2010 Plus, Shimadzu Corporation, Kyoto, Japan) using a flame ionization detector and a capillary column (Zebron ZB-FFAP; 30 m × 0.32 mm × 0.25 μm; Phenomenex Inc., Torrance, CA, USA). The sample injection volume was 1.0 μL, and helium was used as the carrier gas. The column temperature was 110^°^C and the injector and detector temperatures were 250 and 350^°^C, respectively. To determine the concentrations of SCFAs in the samples, the peak heights of the samples were compared to those of actual standards.

### Bioinformatics analysis

Sequencing data were demultiplexed and converted into FASTQ files, and the paired-end sequences were imported into QIIME 2 (Bolyen et al., 2019). The non-biological nucleotides were removed, and sequences were denoised, dereplicated, and chimera-filtered using DADA2 (Callahan et al., 2016). A pre-trained naive Bayes classifier, trained on the SILVA 138 SSU database, was used to assign taxonomies to the sequences, and reads were classified by taxon using the fitted classifier (Quast et al., 2012). For alpha and beta diversity analyses, all samples were rarefied to a common sequencing depth of 40,000 sequences per sample. The alpha diversity indices computed were the number of observed features (number of ASVs), the Shannon diversity index, and Pielou’s evenness. Beta diversity was assessed using Jaccard distances, applying principal coordinate analysis, and generating visualization plots with EMPeror (Vázquez-Baeza et al., 2013). To make predictions about the metabolic functions of the microbial community, Phylogenetic Investigation of Communities by Reconstruction of Unobserved States (PICRUSt2) was used (Douglas et al., 2020). The MetaCyc pathway database was used to identify the metagenome metabolic functions.

### Statistical analysis

A one-way ANOVA was conducted to analyze the effects of the subclinical dose of FUM + DON on dependent variables: body weight gain, FCR, total IgA, *C. perfringens*-specific IgA, and SCFA, with the pen being considered the experimental unit. When there were significant differences, the means were separated using the Student’s *t*-test (JMP Pro 15 software, Cary, NC, USA). Relative bacterial abundance and relative predicted microbial function were analyzed in R version 4.2.0 (R Core Team, 2013). Individual taxa and alpha diversity indexes were analyzed using the Kruskal-Wallis H Test. Comparisons were performed for overall differences between the diets and for differences between days, and contrasts between diets within each timepoint were carried out. Results from all statistical tests were corrected by the Benjamini-Hochberg False Discovery Rate (FDR) procedure for multiple comparisons to limit the number of false positive results. The FDR adjusted *p*-values were statistically significant when *P* ≤ 0.05; and considered a trend when *P*-values were between 0.05 and 0.10.

## Results

### Effect of subclinical dose of FUM + DON on production performances

There were significant (*P* < 0.05) treatment effects on BWG at d 14 and 21 (Table 1). On d 14 and 21, birds in the FUM + DON treatment group had significantly lower BWG (*P* < 0.05) compared to the birds in the control group. On 21 days of age, birds in the FUM + DON group had a 55.1 g lower BWG than the birds in the control group. There was a trend on BWG at d 28 (*P* = 0.07) and d 35 (*P* = 0.06), and FUM + DON decreased the BWG by 65.8 g on d 28 and 84.3 g on d 35.

**TABLE 1:** Effect of subclinical fumonisins (FUM) and deoxynivalenol (DON) contamination on production parameters.

	Parameter	Control	FUM + DON + CP	SEM	*P*-value
D7	Body weight gain (g)	92.0	86.1	2	0.07
	FCR	1.49	1.56	0.07	0.5
D14	Body weight gain (g)	295.4^a^	256.9^b^	8	0.01
	FCR	1.47^b^	1.67^a^	0.04	0.03
D21	Body weight gain (g)	687.2^a^	631.8^b^	13	0.01
	FCR	1.38^b^	1.46^a^	0.05	0.04
D28	Body weight gain (g)	1172.9	1107.1	24	0.07
	FCR	1.43	1.48	0.01	0.1
D35	Body weight gain (g)	1716.8	1632.5	30	0.06
	FCR	1.47	1.5	0.03	0.8
0–35	Mortality (%)	0	5.0	0.3	0.005

Broilers were fed control or mycotoxin (FUM 3 mg/kg diet + DON 4 mg/kg diet). All birds received 1 × 10^8^ CFU/bird of *C. perfringens* on d 19 and 20.

Body weight was measured on days 0, 7, 14, 21, 28, and 35 of age to calculate body weight gain (Table 1).

Feed consumption was measured on days 0, 7, 14, 21, 28, and 35 of age to calculate feed consumption ratio (Table 1).

Mortality-corrected body weight gain and feed conversion ratio is presented. Superscript a and b denote statistically significant differences within a row (P < 0.05) n = 8 pens of 15 birds/pen.

There were significant (*P* < 0.05) treatment effects on the FCR on days 14 and 21 (Table 1). On d 14, birds in the FUM + DON group had a 21 point (*P* = 0.05) higher in FCR compared to the birds in the control group. On d 21, birds in the FUM + DON groups had an eight point significant increase in FCR compared to the birds in the control group.

### Effect of subclinical dose of FUM + DON on bile anti-clostridium IgA after *C. perfringens* challenge

**FIGURE 1:**
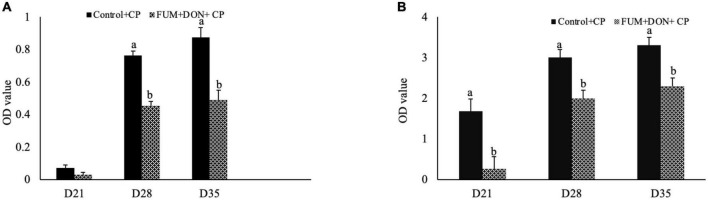
Effect of subclinical fumonisins (FUM) and deoxynivalenol (DON) contamination on bile anti-Clostridium IgA content. Broilers were fed control or mycotoxin (FUM 3 mg/kg diet + DON 4 mg/kg diet). All birds received 1 × 10^8^CFU/bird of *C. perfringens* on d 19 and 20. At 1, 7, and 14 days after infection, bile was analyzed for anti-*C. perfringens* specific IgA **(A)** and total IgA **(B)** by ELISA and expressed as optical density (OD). Bars (+SEM) with “a” and “b” superscript differ significantly (*P* < 0.05).

Bile anti-Clostridium-specific IgA antibody concentration were in the detectable range in both the control and treatment groups at d 21 of age (Figure 1A). There were significant differences between the control and FUM + DON treatment groups on anti-Clostridium–specific bile IgA antibody levels at d 28 and 35 after *C. perfringens* infection. Birds in the FUM + DON group had significantly lower (*P* < 0.05) antibody titers for anti-Clostridium IgA. There were significant differences between the control and FUM + DON treatment groups on total bile-IgA antibody concentration at d 21, 28, and 35 (Figure 1B). Birds in the FUM + DON group had significantly lower (*P* < 0.05) antibody concentrations for total bile IgA.

### Effect of subclinical dose of FUM + DON on intestinal lesion score and ileum histomorphology

**FIGURE 2:**
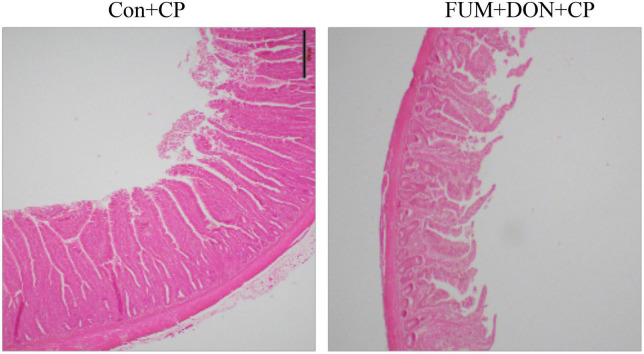
Effect of subclinical fumonisins (FUM) and deoxynivalenol (DON) contamination on Ileum histomorphology at d 21. Broilers were fed control or mycotoxin (FUM 3 mg/kg diet + DON 4 mg/kg diet). All birds received 1 × 10^8^CFU/bird of *C. perfringens* on d 19 and 20 (*n* = 8). Haematoxylin and eosin staining; Scale bar = 500 μm.

There were significant (*p* < 0.05) treatment effects on the NE lesion score on day 21 (Table 2). Subclinical dose of FUM + DON increased (*p* < 0.05) the Wilcoxon/Kruskal–Wallis Score Means for lesion scores compared with the control group on day 21. Additionally combined FUM + DON increased the necrosis at the tip of the villi (Figure 2) and significantly increased the intestinal lesion score (*P* < 0.05).

**TABLE 2:** Effect of subclinical fumonisins (FUM) and deoxynivalenol (DON) contamination on necrotic enteritis lesion scores.

Treatment	Score 0	Score 1	Score 2	Score 3	Rank scores mean	Chi sq. *p*-value
Control + CP	23	1	0	0	19.5	0.01
FUM + DON + CP	13	11	0	0	29.5	–

Broilers were fed control or mycotoxin (FUM 3 mg/kg diet + DON 4 mg/kg diet). All birds received 1 × 10^8^CFU/bird of *C. perfringens* on d 19 and 20. On day 21, three birds/pen (*N* = 24) were scored for necrotic enteritis lesion scores on a 0–3 scale wherein 0 is normal, 1 shows slight mucus covering the small intestine, 2 has a necrotic small intestinal mucosa, and 3 shows sloughed cells and blood in the small intestinal mucosa and contents. Lesion scores were analyzed by a non-parametric test, and Wilcoxon/Kruskal–Wallis rank-sum test was used to separate the means.

### Effect of subclinical dose of FUM + DON on the cecal microbiota

**FIGURE 3:**
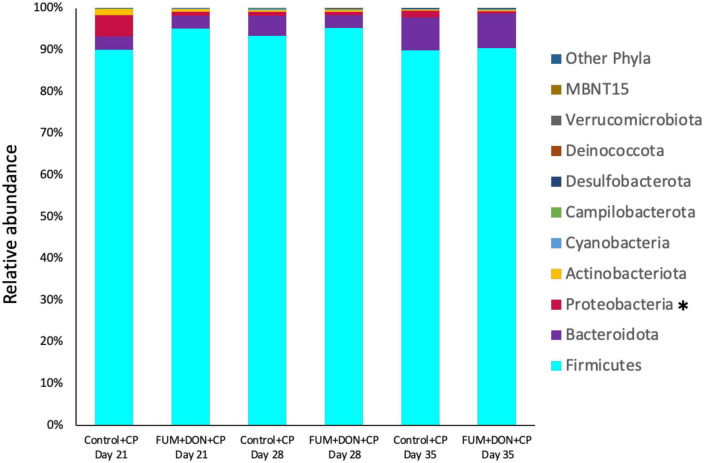
Effect of subclinical fumonisins (FUM) and deoxynivalenol (DON) contamination on relative abundance of microbiome composition at phylum level. Broilers were fed control or mycotoxin (FUM 3 mg/kg diet + DON 4 mg/kg diet). All birds received 1 × 10^8^CFU/bird of *C. perfringens* on d 19 and 20. Cecal digesta was analyzed for microbiome composition at phyla level using 16S rRNA sequencing at 1, 7, and 14 days post-infection. Asterisk denotes significant difference (*P < 0.05).

**FIGURE 4:**
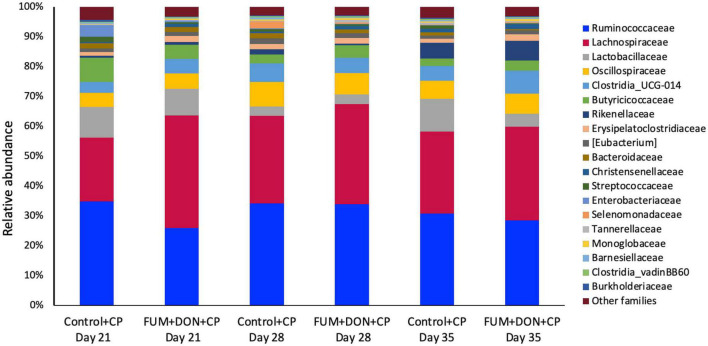
Relative abundance of microbiome at the family level. Broilers were fed control or mycotoxin (FUM 3 mg/kg diet + DON 4 mg/kg diet). All birds received 1 × 10^8^CFU/bird of *C. perfringens* on d 19 and 20. Cecal digesta was analyzed for relative abundance of microbiome using 16S rRNA sequencing at 1, 7, and 14 days post-infection.

**FIGURE 5:**
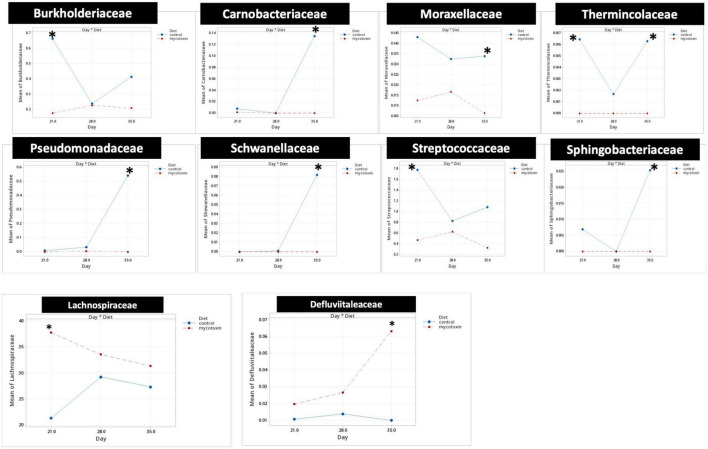
Bacterial families found to be significantly different (*P* < 0.05) between treatments during the study. Broilers were fed control or mycotoxin (FUM 3 mg/kg diet + DON 4 mg/kg diet). All birds received 1 × 10^8^CFU/bird of *C. perfringens* on d 19 and 20. Cecal digesta was analyzed for microbiome composition at family level using 16S rRNA sequencing at 1, 7, and 14 days post-infection. Values are presented as least squares means *n* = 8 (Kruskal-Wallis tests corrected by FDR). Asterisks denote significant difference. *P < 0.05.

At the phylum level, *Firmicutes* (92.0%), *Bacteroidota* (5.0%), and *Proteobacteria* (1.7%) were the most predominant phyla in the cecal content (Figure 3). The abundance of *Proteobacteria* was significantly lower (*P* = 0.001) in the FUM + DOM group compared to the control. At the family level, the most abundant microbial community was *Ruminococcaceae* (31.0%), followed by *Lachnospiraceae* (30.2%), *Lactobacillaceae* (6.8%), *Oscillospiraceae* (6.4%), *Clostridia* UCG-014 (5.4%), and *Butyricicoccus* (4.3%) (Figure 4). *Burkholderiaceae*, *Carnobacteriaceae, Moraxellaceae, Pseudomonadaceae, Pseudonocardiaceae, Shewanellaceae, Sphingobacteriaceae, Streptococcaceae*, and *Thermincolaceae* relative abundances were all decreased (P 0.05) in the FUM + DON group (Figure 5). Conversely, combined FUM and DON significantly increased the relative abundances of *Defluviitaleaceae* by 5.3-fold and *Lachnospiraceae* by 0.15-fold (*P* = 0.05) compared to the control group on d 35 (Figure 5). At the genus level, the predominating microbial community were *Faecalibacterium* (19.9%), *Ruminococcaceae* (13.6%), *Lactobacillus* (6.8%), and *Butyricicoccus* (4.2%) (Supplementary Figure 1). On d 35, FUM + DON contamination decreased the *Acinetobacter* by 0.5-fold and *Pseudomonas* by 0.8-fold (*P* ≤ 0.05) in the cecal content, and FUM + DON contamination abundance tended (*P* = 0.08) to be decreased for *Thermincola* (Supplementary Figure 2).

**FIGURE 6:**
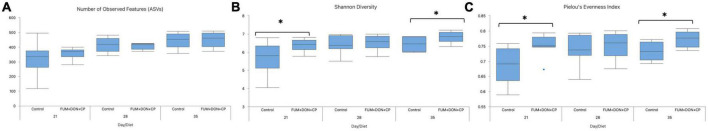
Effect of subclinical fumonisins (FUM) and deoxynivalenol (DON) contamination on Alpha-Diversity indexes: Number of observed features **(A)**, Shannon diversity **(B)**, and Pielou’s evenness index **(C)**. Broilers were fed control or mycotoxin (FUM 3 mg/kg diet + DON 4 mg/kg diet). All birds received 1 × 10^8^ CFU/bird of *C. perfringens* on d 19 and 20. Cecal digesta was analyzed for microbiome composition using 16S rRNA sequencing at 1, 7, and 14 days post-infection. Significance of day for number of observed features (*P* < 0.001), Shannon diversity (*P* = 0.02), Pielou’s evenness (*P* = 0.29). Significance of diet for number of observed features (*P* = 0.85), Shannon diversity (*P* = 0.04), Pielou’s evenness (*P* = 0.003). Asterisks denote significant difference (*P < 0.05).

For both diets, microbial richness and diversity increased (*P* ≤ 0.05) from d 21 to 35 (Figure 6). There were no differences between treatments regarding the number of observed features (ASVs) (*P* = 0.85); however, FUM + DON significantly increased the microbial diversity (*P* ≤ 0.05) and evenness (*P* ≤ 0.05) compared to the control groups.

**FIGURE 7:**
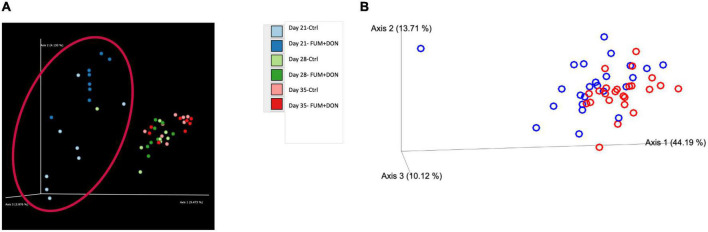
Effect of subclinical fumonisins (FUM) and deoxynivalenol (DON) contamination on **(A)**. Beta-diversity [based on Jaccard distances and **(B)**]. Principal coordinate analysis with Bray-Curtis dissimilarity of the gut microbial community structure. Broilers were fed control or mycotoxin (FUM 3 mg/kg diet + DON 4 mg/kg diet). All birds received 1 × 10^8^CFU/bird of *C. perfringens* on d 19 and 20. Cecal digesta was analyzed for microbiome composition using 16S rRNA sequencing at 1, 7, and 14 days post-infection. Significant differences were observed between two groups by PERMANOVA test (*P* = 0.001).

The distribution of the stabilization control and the FUM + DON samples were explored *via* Jaccard distances (Figure 7A) and Principal Coordinates Analysis (PCoA) with Bray–Curtis distance (Figure 7B). As depicted Figure 7, cecal microbiome profile from FUM + DON groups were well-separated on d 21.

### Effect of subclinical dose of FUM + DON on short chain fatty acids

**FIGURE 8:**
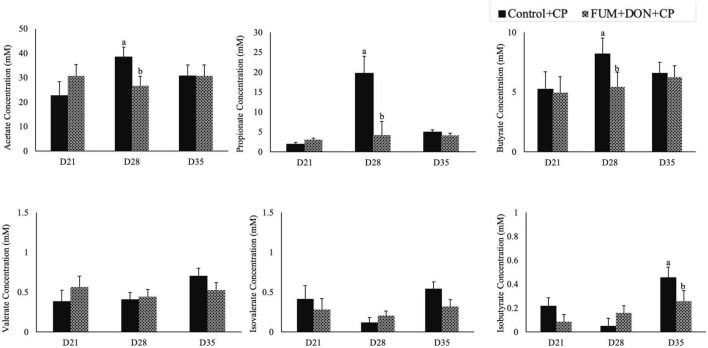
Effect of subclinical fumonisins (FUM) and deoxynivalenol (DON) contamination on of short chain fatty acids (SCFA). Broilers were fed control or mycotoxin (FUM 3 mg/kg diet + DON 4 mg/kg diet). All birds received 1 × 10^8^CFU/bird of *C. perfringens* on d 19 and 20. Cecal digesta was analyzed SCFA at 1, 7, and 14 days post-infection. Bars (+SEM) with “a” and “b” superscripts indicate statistical differences within an age (*P* < 0.05).

Subclinical doses of FUM + DON significantly decreased the acetate, propionate butyrate concentration on d 28 (*P* < 0.05, Figure 8) and iso butyrate concentration on d 35. Additionally, feeding FUM + DON tended to decrease the iso-valerate concentration in the cecal content on d 35 (*P* = 0.08).

### Correlation analysis

**FIGURE 9:**
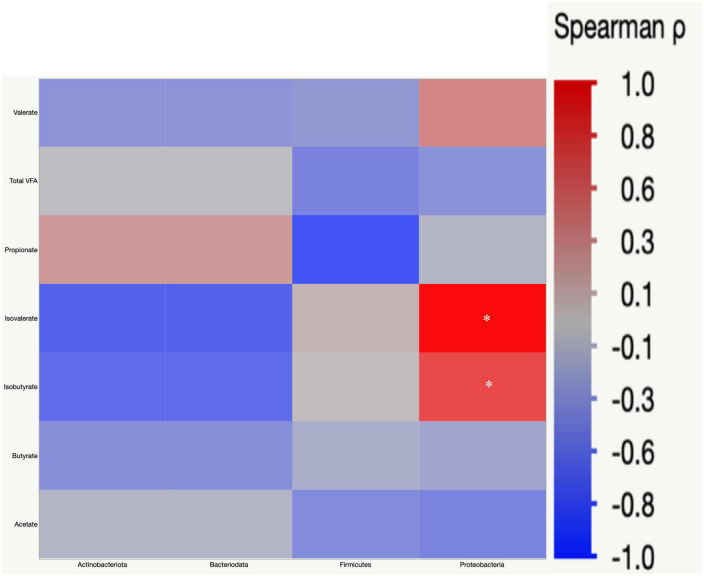
Spearman’s rank correlation matrix of the four main phyla and short chain fatty acids (SCFA). The colors of the scale bar denote the nature of the correlation with one indicating a perfect positive correlation (dark red) and –1 indicating perfect negative correlation (dark blue). Significant correlations are indicated by asterisk (*P < 0.05).

Pearson’s correlation was used to understand the correlation between bacterial abundances, and SCFA concentrations in cecal digesta. *Proteobacteria* had a positive correlation with isovalerate (r = 0.52) and isobutyrate (r = 0.36) (Figure 9).

### Effect of subclinical dose of FUM + DON on functional metagenome prediction

**FIGURE 10:**
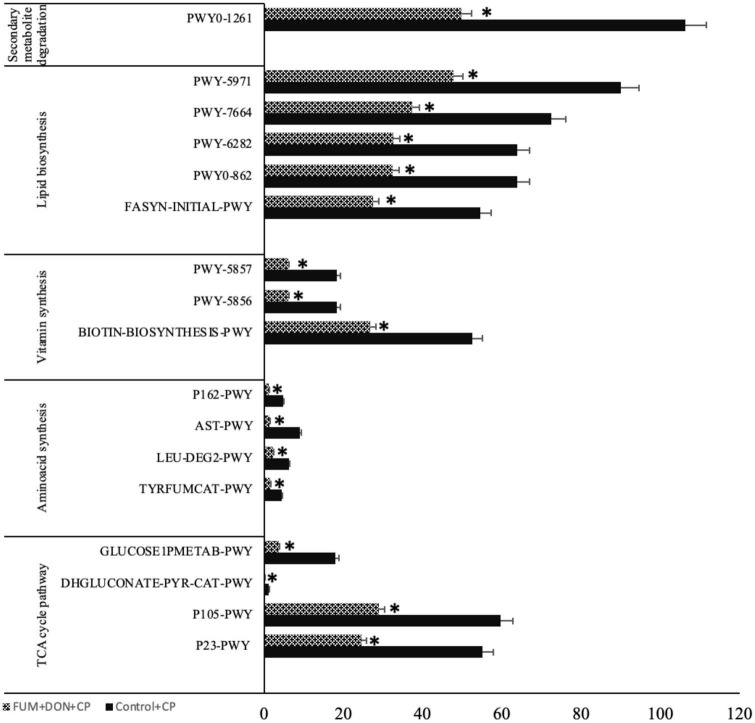
Microbial function analysis: Broilers were fed control or mycotoxin (FUM 3 mg/kg diet + DON 4 mg/kg diet). All birds received 1 × 10^8^CFU/bird of *C. perfringens* on d 19 and 20. Cecal digesta was analyzed SCFA at 1, 7, and 14 days post-infection. P23-PWY: Reductive TCA cycle I; P105-PWY: TCA cycle IV (2-oxoglutarate decarboxylase); DHGLUCONATE-PYR-CAT-PWY: Glucose degradation (oxidative); GLUCOSE1PMETAB-PWY: Glucose and glucose-1-phosphate degradation; TYRFUMCAT-PWY: L-tyrosine degradation I; LEU-DEG2-PWY: L-leucine degradation I; AST-PWY: L-arginine degradation II (AST pathway) (AST-PWY); P162-PWY: L-glutamate degradation V (*via* hydroxyglutarate); BIOTIN-BIOSYNTHESIS-PWY: Biotin Biosynthesis I; PWY-5856: Cofactor, Carrier, and Vitamin Biosynthesis; PWY-5857: Cofactor, Carrier, and Vitamin Biosynthesis; PWY-5971: Palmitate Biosynthesis; PWY-6282: Fatty Acid and Lipid Biosynthesis; PWY-7664: Fatty Acid and Lipid Biosynthesis; FASYN-INITIAL-PWY: Fatty Acid and Lipid Biosynthesis; PWY0-1261: Secondary metabolite degradation. Bars (+SEM) with no common superscript differ significantly. Asterisks denote significant difference (*P < 0.05) *n* = 8.

To evaluate potential modifications in bacterial metabolism driven by dietary FUM + DON, functional metagenome prediction was performed. FUM and DON exposure decreased the metabolic pathways of TCA cycle degradation, fatty acid and lipid biosynthesis, cofactor and vitamin biosynthesis, amino-acid synthesis and secondary metabolites degradation (*P* ≤ 0.05) (Figure 10).

## Discussion

The most frequent mycotoxins found in poultry feed ingredients are FUM and DON (Weaver et al., 2021). Recent research from our lab has demonstrated that broiler productivity and health are adversely impacted by exposure to combined subclinical doses of 3 mg/kg FUM and 4 mg/kg DON (Shanmugasundaram et al., 2022). Because FUM and DON are not efficiently absorbed from the intestine, gut epithelial cells are typically exposed to the ingested toxins at the highest concentration (Grenier et al., 2016). DON and FUM alter intestinal homeostasis and increase the leakage of plasma amino acids into the intestinal lumen, providing a suitable environment for *C. perfringens* proliferation (Antonissen et al., 2014). As a result, several key intestinal functions, like intestinal barrier functions, are compromised, ultimately leading to subclinical necrotic enteritis (Antonissen et al., 2014, 2015). These changes in intestinal physiological functions affect the gut microbiome and can cause bacterial translocation and the onset of infectious diseases corresponding to changes in the health status of chickens (Hills et al., 2019). However, very limited information is available on the impacts of the chicken gut microbiota on combined mycotoxins and the effects of mycotoxins on the gut microbiome. The objective of this study was to determine if combined FUM and DON cause cecal microbiota dysbiosis and, hence, can explain the induction of subclinical necrotic enteritis.

In the present study, the combined FUM and DON significantly decreased the chicken body weight gain by 39 g and increased the feed conversion ratio by 21 points on d 14. Persistent exposure to multiple mycotoxins at subclinical doses decreased the growth performance of broilers (Young et al., 2007). Similarly, chickens fed contaminated feed containing 20 mg/kg FUM and 1.5 mg/kg DON or 20 mg/kg FUM and 5 mg/kg DON had lower BWG (Guerre, 2020). However, feed contaminated with 5 mg/kg DON alone (Eriksen et al., 2002; Guerre, 2020) or 20 mg/kg (Guerre, 2020) or 50 mg/kg (Zhai et al., 2019) of FUM alone did not negatively affect the body weight gain in broilers. Previous research has identified that DON activates the mitogen-activated protein kinase pathways to inhibit the synthesis of tight junction proteins in enterocytes, resulting in a leaky gut and lower production performance in broilers (Lucioli et al., 2013). In our study, histomorphology analysis of ileum samples on d 21 showed that the combined FUM and DON synergistically damaged the villi structure, resulting in decreased production performance. Villi damage caused by FUM + DON (Figure 3) is likely to reduce nutrient absorption, resulting in decreased production performance (Grenier et al., 2016).

The FUM and DON-induced decrease in body weight was more pronounced at 21 days compared to the decrease in body weight at 35 days. The negative effect of mycotoxins on growth depends on the growth phase of the animals, with younger animals being more susceptible than older animals. A meta-analysis study with pigs identified that including the age of pigs as a covariable improved the estimation of mycotoxin effects and suggested that the increased sensitivity of young animals to mycotoxins is likely because of the reduced detoxification enzymes in the liver of young animals (Andretta et al., 2012). In addition, younger birds have a lower microbial alfa diversity (Lundberg et al., 2021), which can be expected to decrease the ability of gut microbes to detoxify mycotoxin in young broilers.

In broilers, exposure to DON and FUM can compromise mucosal immunity (Bracarense et al., 2012). In the current study, the combined DON and FUM decreased the concentration of total IgA and *C. perfringens*-specific IgA in bile. These results are in agreement with the previous study: 10 mg DON/kg suppressed the production of antibodies against the infectious bronchitis vaccine (IBV) (Ghareeb et al., 2012) and to the Newcastle disease virus (NDV) in broilers (Danicke et al., 2002). Given that B cells and T cells are highly proliferative cells with high protein turnover, combined FUM and DON can be expected to target B cells and reduce antibody production.

The exposure to a combined subclinical dose of FUM and DON increased microbial evenness and diversity at days 21 and 35. In the current study, FUM and DON increased the alpha-diversity of the cecal microbiome despite having a negative impact on body weight, feed consumption, and intestinal lesion scores. It is possible that some taxa within the bacterial community may have had growth benefits leading to an increase in the diversity of the microbial species, as indicated by the greater Shannon index values in the FUM + DON group. Chickens were exposed to FUM and DON from their very first day of life, thereby modifying the initial bacterial colonization population in the ceca (Lucke et al., 2018). Even though it is believed that a microbiome with a high species diversity is more stable and less susceptible to disease invasion, a bacterial population that is less diversified and more specialized would still be beneficial to the host because it would make better use of the available resources and promote the host’s synthesis of energy (Hibbing et al., 2010). It can therefore be concluded that even though FUM and DON increased the species evenness and diversity in the cecal microbiota, the microbiota as a whole failed to prevent the induction of subclinical necrotic enteritis. Further significant differences on Jaccard dissimilarity and Principal coordinates analysis (PCoA) with Bray-Curtis dissimilarity of the gut microbial community structure indicated that combined FUM + DON, significantly affected the gut microbiome diversity.

Gut microbiota are the host’s first line of defense against toxic metabolites in feed (Apajalahti and Vienola, 2016). The host gut microbes have the ability to detoxify or transform mycotoxins by secreting enzymes into the gut. Most of the transformation involved the hydrolysis of mycotoxins and the formation of metabolites that are less toxic than the parent compound (Coleman et al., 1997). In chickens, gut microbiota can de-epoxidation DON to DOM-1 (Young et al., 2007); however, this mechanism is less efficient in chickens. Further, the recovery rate of high concentrations of fumonisins from the different parts of the intestinal tract and the excreta of birds fed a FB-contaminated diet indicates that chicken gut microbes are less efficient at hydrolyzing the fumonisins (Guerre, 2015; Grenier et al., 2017). Several bacterial species belonging to the phylum *Proteobacteria* have been recognized for their mycotoxin-detoxifying ability (Zhai et al., 2019). In this study, exposure to FUM and DON decreased (*P* = 0.001) the relative abundance of *Proteobacteria*. Similar results were observed when continuous exposure to 10 mg/kg DON decreased the relative abundance of *Proteobacteria* (Lucke et al., 2018). The decrease in the phyla of *Proteobacteria* was reflected in the reduction in the relative abundance of *Pseudomonadaceae*, *Moraxellaceae*, and *Shewanellaceae* at day 35. The decrease in the relative abundance of Proteobacteria was accompanied by an increase in *Firmicutes* at d 21 and 35. Combined FUM and DON increased the *Firmicutes* to *Proteobacteria* ratio toward more *Firmicutes*, indicating that combined doses of FUM and DON, even at subclinical doses, may contribute to intestinal dysbiosis.

The decrease in the relative abundance of the *Pseudomonadaceae* family was reflected in the decrease in the relative abundance of the *Pseudomonas* genera at day 35. *Pseudomonas* spp. are non-pathogenic gut bacteria that play an essential role in producing vitamin B12 (Balabanova et al., 2021) and biodegrading toxic compounds such as mycotoxins (Wang et al., 2020). In this study, FUM and DON exposure decreased the *Pseudomonas* spp. Thus, the reduction in the relative abundance of the *Pseudomonas* genus might weaken the gut microbiota’s ability to detoxify toxic mycotoxins, a potential cause of necrotic enteritis.

Fumonisins and DON decreased the relative abundance of the *Moraxellaceae* family, which includes the genus *Acinetobacter*. *Acinetobacter* species produce extracellular enzymes that oxidize zearalenone (Yu et al., 2011) and convert DON into the less harmful 3-epimer-DON metabolite (Wilson et al., 2017). The reduction in the relative abundance of the *Acinetobacter* genus might increase the broiler’s susceptibility to a wide array of mycotoxins. Similarly, the *Shewanellaceae* family, which contains *Shewanella* as the only genus (MacDonell and Colwell, 1985), decreased in broilers exposed to FUM and DON. *Shewanella* bacteria is a facultative anaerobe that can utilize heavy metals and toxic compounds as electron donors and detoxify heavy metals and toxic compounds (Thorell et al., 2019). Furthermore, *Shewanella algae strain YM8* produces antifungal compounds to inhibit the growth of *Aspergillus* and the production of aflatoxins (Gong et al., 2015). The decrease in the relative abundance of the *Shewanellaceae* family likely led to a decrease in the mycotoxin-detoxifying ability of the cecal microbiota, thereby leading to the induction of intestinal dysbiosis.

Fumonisins and DON decreased the relative abundance of *Streptococcaceae* and *Carnobacteriaceae*. The *Streptococcaceae* family consists of gram-positive bacteria within the order *Lactobacillales* (Lory, 2014; Rosenberg et al., 2014). Several members of the *Streptococcaceae* family are recognized for their probiotic potential (Lory, 2014). Pigs exposed to combined DON and ZEN for seven days had a significantly decreased relative abundance of *Streptococcaceae* (Le Sciellour et al., 2020). Similarly, mice exposed to 1.5 mg/kg body weight ochratoxin had a decrease in *Streptococcaceae* relative abundance (Izco et al., 2021). Lactic acid bacteria, including *Streptococcus thermophilus*, can bind and neutralize FUM and DON (Niderkorn et al., 2006). The reduction in the relative abundance of the *Streptococcaceae* family can lead to a higher accumulation of mycotoxin, predisposing birds to necrotic enteritis.

Fumonisins and DON exposure increased the relative abundance of the *Lachnospiraceae* family. However, FB1 or combined DON and ZEN have been identified to decrease *Lachnospiraceae* and increase *Lactobacillaceae* (Mateos et al., 2018). The role of the *Lachnospiraceae* family in gut health is not clearly defined (Vacca et al., 2020). *Lachnospiraceae, Lactobacillaceae*, and *Ruminococcaceae* species of *Firmicutes* hydrolyze starch and other carbohydrates to produce butyrate and other SCFAs, which can be expected to improve gut health in birds. Even though FUM and DON significantly increased the *Lachnospiraceae* abundance at days 21, 28, and 35, the predicted metagenomic pathway analysis identified that FUM and DON impaired carbohydrate metabolism, potentially contributing to necrotic enteritis. High relative abundances of *Lachnospiraceae* were positively correlated with impaired glucose and lipid metabolism and decreased SCFA, indicating metabolic disturbance in this study. Bacterial strains belonging to the family *Lachnospiraceae* are associated with metabolic diseases such as IBD, obesity, and liver diseases (Vacca et al., 2020). However, several members of the *Lachnospiraceae* family play a significant role in maintaining the health and homeostasis of the gut. *Roseburia intestinalis*, a member of the *Lachnospiraceae* family, produces butyrate to improve the integrity of the intestinal barrier (Kasahara et al., 2018). It is likely that the increase in the *Lachnospiraceae* family in FUM and DON-exposed birds was a gut bacteria-host interaction response to aid the recovery of damaged intestinal cells by producing SCFAs to promote the growth performance.

On d 35, exposure to FUM and DON decreased the relative abundance of the *Lactobacillus* and *Faecalibacterium* genera while increasing the *Clostridia (uncultured genus level group) UCG-014*. In contrast, piglets exposed to fumonisin B1 increased *Lactobacillus* and decreased *Faecalibacterium* (Mateos et al., 2018). In pigs, 47% of fumonisins were hydrolyzed into less toxic metabolites, as evidenced by the increased relative abundance of the *Lactobacillus* genus (Schertz et al., 2018). *Lactobacillus* produces numerous proteolytic enzymes that play a critical role in the detoxification of mycotoxins. The *Lactobacillus* metabolizes the FUM by using polysaccharides and peptidoglycans on their bacterial cell wall to bind to the mycotoxins and increase their physical adsorption (Zhao et al., 2016). Therefore, the reduction in the relative abundance of *Lactobacillus* in chickens is most likely related to their disintegrated binding sites for FUM and, as a result, their decreased ability to metabolize the toxins. The relative abundances of *Faecalibacterium* and *Clostridiaceae* indicate that FUM and DON enhanced the inflammation and provided a favorable environment for *Clostridia UCG-014* as a consequence of intestinal dysbiosis, and this most likely contributes to subclinical necrotic enteritis.

One of the limitations of this study is that all birds received *C. perfringens* inoculation, and hence the interaction between *C. perfringens* and FUM and DON could be a confounding factor in this study. Given that *C. perfringens* is commensal in the poultry intestine and ubiquitous in the poultry environment, this interaction could represent a real-world scenario in which feed FUM, and DON interact with toxins produced by *C. perfringens*, exacerbating gut damage.

In conclusion, our data suggest that exposure to FUM and DON alters the cecal microbiota in birds and induces intestinal dysbiosis, and ultimately increasing the risk of subclinical necrotic enteritis. The decrease in the relative abundance of bacterial families involved in the biodegradation of FUM and DON exacerbates the bird’s susceptibility to the toxins produced by *C. perfringens*. Our study demonstrated that there is a direct relationship between microbiota, mycotoxins, and host performance. Continuous exposure to multiple mycotoxins, even at subclinical doses, had a negative effect on microbiota composition and microbiota-related metabolic functions. FUM and DON, even at a subclinical dose, dysregulate intestinal functions, impairing the gut immune response, which eventually has the potential to predispose the birds to necrotic enteritis. The effect of mycotoxin within the gut varies considerably due to bioavailability, excretion, the effect of the microbiota on mycotoxins, and which segment of the gut is being analyzed. Future studies analyzing the different parts of the gut microbiome profile in response to FUM, and DON exposure would provide a clear picture of the impact of mycotoxins on the gut microbiome.

## Data availability statement

The sequencing data is publicly available on the MG-RAST website (www.mg-rast.org) under accession number: mgm4991309.3.

## Ethics statement

This animal study was reviewed and approved by Institutional Animal Care and Use Committee at the Southern Poultry Research Group, Athens, GA.

## Author contributions

RS: conceptualization, investigation, methodology, data curation, writing—original draft preparation, and funding acquisition. JL: methodology, data curation, and writing—review and editing. WH and MD: methodology and writing—review and editing. TA: writing—review and editing. All authors contributed to the article and approved the submitted version.
