# Supplementary material for: Subclinical doses of dietary fumonisins and deoxynivalenol cause cecal microbiota dysbiosis in broiler chickens challenged with Clostridium perfringens
Source: Front Microbiol. 2023 Apr 3;14:1106604. doi: 10.3389/fmicb.2023.1106604 (PMC10111830; doi:10.3389/fmicb.2023.1106604)
Supplement: Supplementary file 1 [file Presentation_1.pptx]

## Slide 1
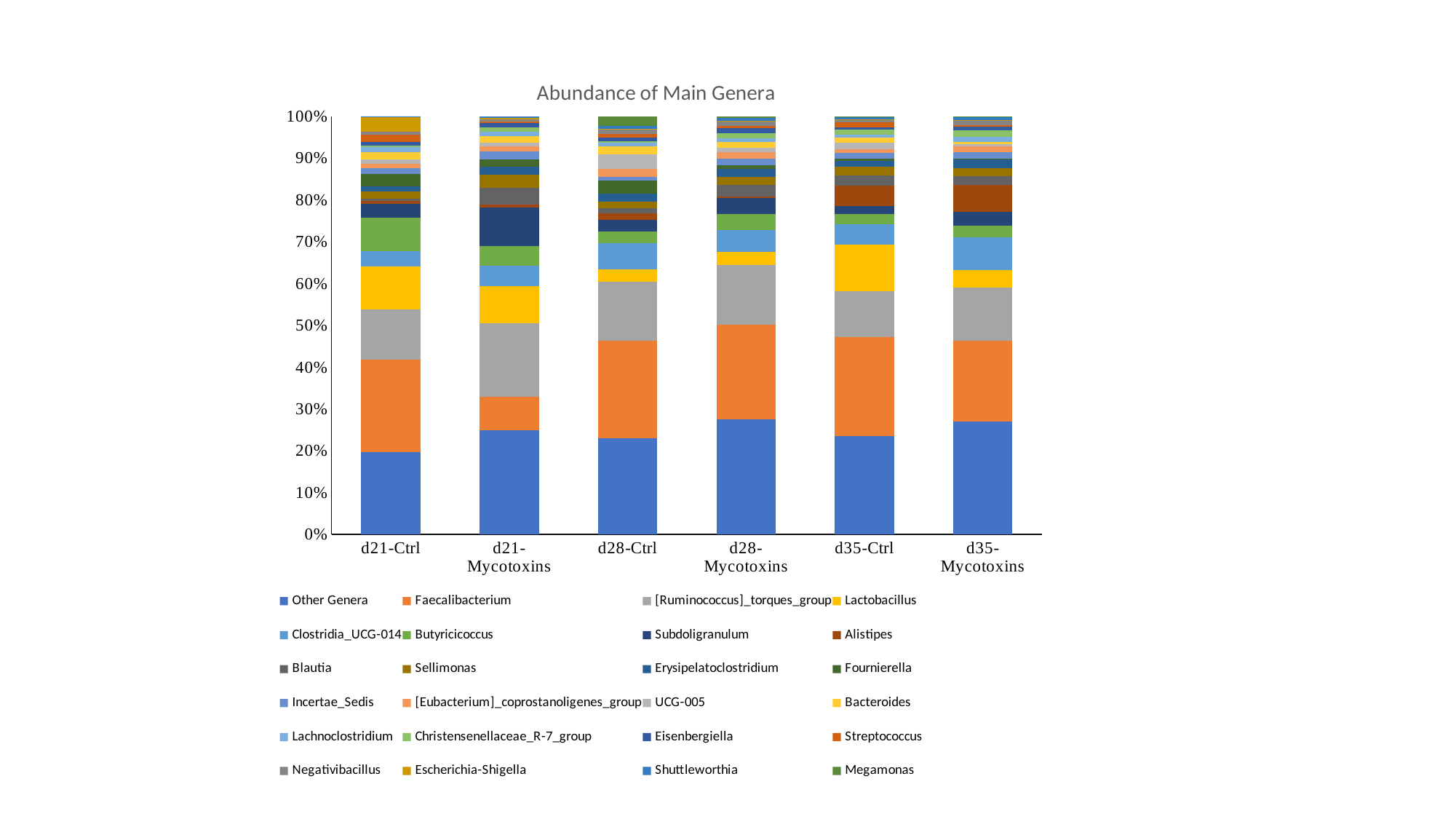

### Chart: Abundance of Main Genera
| Category | Other Genera | Faecalibacterium | [Ruminococcus]_torques_group | Lactobacillus | Clostridia_UCG-014 | Butyricicoccus | Subdoligranulum | Alistipes | Blautia | Sellimonas | Erysipelatoclostridium | Fournierella | Incertae_Sedis | [Eubacterium]_coprostanoligenes_group | UCG-005 | Bacteroides | Lachnoclostridium | Christensenellaceae_R-7_group | Eisenbergiella | Streptococcus | Negativibacillus | Escherichia-Shigella | Shuttleworthia | Megamonas |
|---|---|---|---|---|---|---|---|---|---|---|---|---|---|---|---|---|---|---|---|---|---|---|---|---|
| d21-Ctrl | 19.715674230500014 | 22.084008173375 | 12.025729880125 | 10.244551684 | 3.587430247625 | 7.97404015325 | 3.305878087 | 0.7283886386249999 | 0.585224231125 | 1.7277729342500003 | 1.23901955425 | 3.014036650125 | 1.259002123875 | 1.181621349625 | 0.8952484213750002 | 1.77015715325 | 1.221808608875 | 0.39388102525000007 | 0.923571947875 | 1.7637987038750003 | 0.53794750525 | 3.5824477553749996 | 0.237356972625 | 0.0014039685 |
| d21-Mycotoxins | 24.93597680137502 | 7.994785867875001 | 17.566943562625 | 8.888487647125 | 4.844657204875 | 4.757467746874999 | 9.152636958625 | 0.7231868692499999 | 4.057245354375 | 3.088662862125 | 1.9433734289999998 | 1.62297860875 | 1.8988911178749999 | 1.372682123625 | 0.7106203204999999 | 1.630277724875 | 0.9855260617500001 | 1.14004243175 | 0.9594414813750001 | 0.462727349625 | 0.406126600125 | 0.3685204685 | 0.488741407125 | 0.0 |
| d28-Ctrl | 22.981252838875008 | 23.369531896125 | 13.977698478749998 | 3.0908775732500002 | 6.147853395375 | 2.792858614125 | 2.751024475625 | 1.5656837596250002 | 1.310402488375 | 1.6299709511250002 | 1.785653829625 | 3.1250274686250004 | 0.9654439408750001 | 1.840094296875 | 3.51283555725 | 1.84085159875 | 0.899553647375 | 0.453486625375 | 0.846080707875 | 0.8119136634999999 | 1.068991784125 | 0.17828919062499998 | 0.6374308306250001 | 2.41719238725 |
| d28-Mycotoxins | 27.412070178499988 | 22.6602438835 | 14.246084408249999 | 3.296994342625 | 5.088149236 | 3.8894149054999994 | 3.755878414625 | 0.451921366875 | 2.7934384994999997 | 1.8214078947499999 | 1.8956933644999998 | 0.981874119625 | 1.57429711175 | 1.5617578426249998 | 1.07381177475 | 1.265136431375 | 0.97345349325 | 1.1393057431250002 | 1.239468881375 | 0.617237490625 | 1.044972943875 | 0.15112060300000002 | 0.7034512777499999 | 0.36281579224999994 |
| d35-Ctrl | 23.436989214375004 | 23.735583257625 | 11.016913685875 | 11.05898571225 | 4.950665969125 | 2.390523925875 | 1.9929339405 | 4.8673931692500005 | 2.3224792993750003 | 2.1109752847500003 | 1.3806146385 | 0.485345912125 | 1.4306288815 | 0.9864647223749999 | 1.529770637625 | 1.1624744065 | 0.749489827375 | 1.188279291375 | 0.572699380625 | 1.07449974875 | 0.6868392105 | 0.16101894200000003 | 0.656444493 | 0.05198644875 |
| d35-Mycotoxins | 26.99548171437499 | 19.286868106375 | 12.669063632875002 | 4.276805910375 | 7.703830114000001 | 2.937231910625 | 3.22269857175 | 6.522615709249999 | 2.07379547575 | 1.827178475625 | 2.082068095125 | 0.18261902075000003 | 1.65668174625 | 1.3944274648750001 | 0.545355697875 | 0.43580969500000005 | 1.218047378 | 1.53060079925 | 0.9021040502500001 | 0.32458853887499994 | 1.16866939275 | 0.06608181925 | 0.6618818077499999 | 0.315494873 |
