# Supplementary material for: Subclinical doses of dietary fumonisins and deoxynivalenol cause cecal microbiota dysbiosis in broiler chickens challenged with Clostridium perfringens
Source: Front Microbiol. 2023 Apr 3;14:1106604. doi: 10.3389/fmicb.2023.1106604 (PMC10111830; doi:10.3389/fmicb.2023.1106604)
Supplement: Supplementary file 2 [file Presentation_2.pptx]

## Slide 1
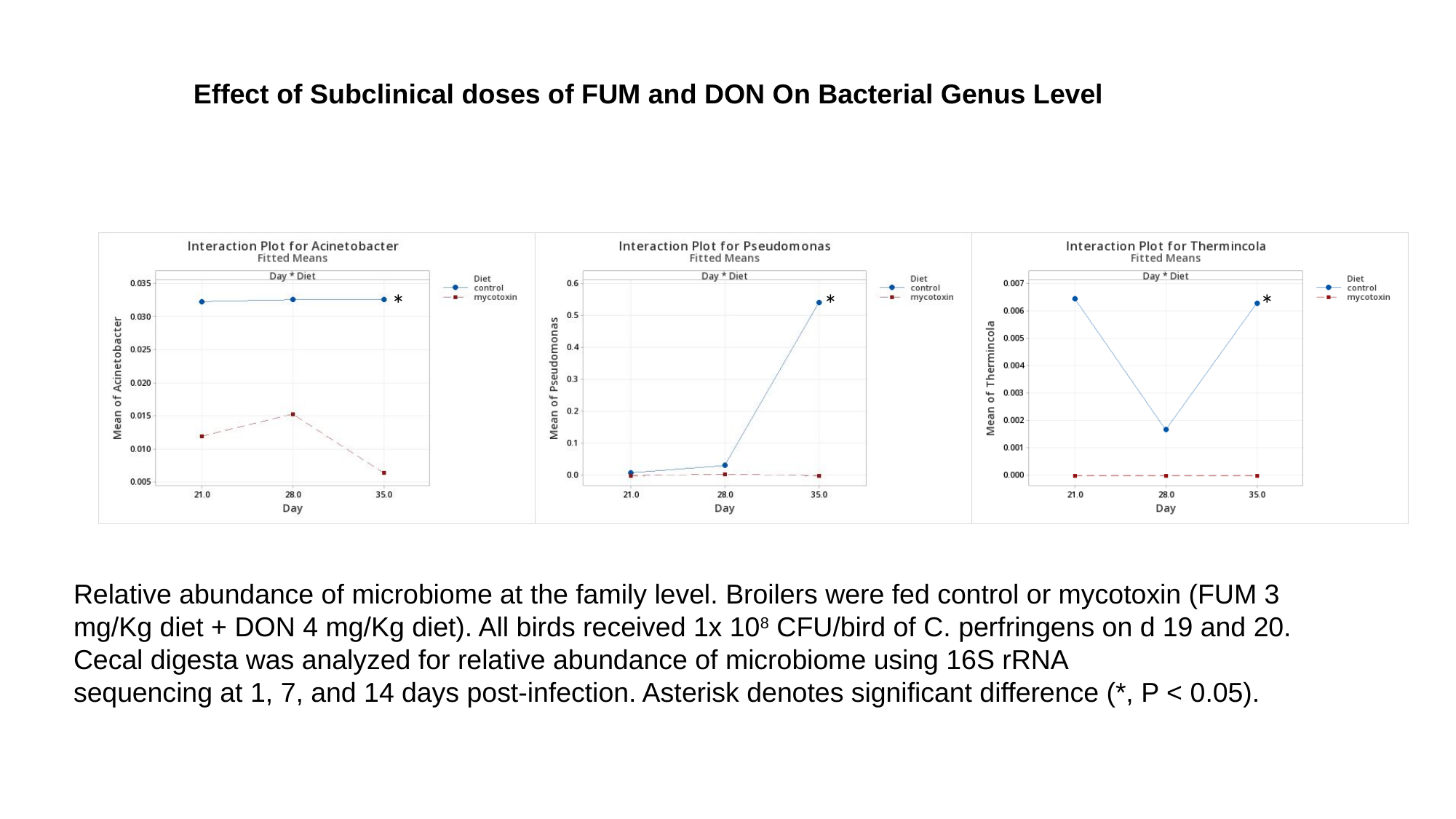

Effect of Subclinical doses of FUM and DON On Bacterial Genus Level
*
*
*
Relative abundance of microbiome at the family level. Broilers were fed control or mycotoxin (FUM 3 mg/Kg diet + DON 4 mg/Kg diet). All birds received 1x 10^8^ CFU/bird of C. perfringens on d 19 and 20. Cecal digesta was analyzed for relative abundance of microbiome using 16S rRNA
sequencing at 1, 7, and 14 days post-infection. Asterisk denotes significant difference (*, P < 0.05).
