# Supplementary material for: Subclinical doses of dietary fumonisins and deoxynivalenol cause cecal microbiota dysbiosis in broiler chickens challenged with Clostridium perfringens
Source: Front Microbiol. 2023 Apr 3;14:1106604. doi: 10.3389/fmicb.2023.1106604 (PMC10111830; doi:10.3389/fmicb.2023.1106604)
Supplement: Supplementary file 3 [file Table_1.docx]

**Supplementary Table 1. Ingredient and nutrient composition of basal-diets (as-fed basis)**

| **^1^Ingredients (%)** | **Starter** | **Finisher** |
| --- | --- | --- |
| Corn | 56.29 | 64.86 |
| Soybean meal, 48% CP | 37.87 | 28.44 |
| Soybean oil | 2.18 | 3.80 |
| Dicalcium phosphate | 1.48 | 0.84 |
| Calcium carbonate | 0.91 | 0.78 |
| Sodium chloride | 0.40 | 0.40 |
| MHA | 0.37 | 0.32 |
| L-Lysine | 0.21 | 0.22 |
| Trace Mineral Premix | 0.10 | 0.10 |
| Choline Chloride (60%) | 0.07 | 0.08 |
| L-Threonine | 0.06 | 0.07 |
| Vitamin Premix | 0.05 | 0.05 |
| Phytase (500 ftu) | 0.01 | 0.01 |

Nutrients, vitamins and minerals were provided in the form and amount described in the NRC Standard Reference Diet for chickens (NRC, 1994).

^1^ Supplied per kilogram of diet: vitamin A, 5,511 IU; vitamin D3, 1,102 ICU; vitamin E, 11.02 IU; vitamin B12, 0.01 mg; biotin, 0.11 mg; menadione, 1.1 mg; thiamine, 2.21 mg; riboflavin, 4.41 mg; d-pantothenic acid, 11.02 mg; vitamin B6, 2.21 mg; niacin, 44.09 mg; folic acid, 0.55 mg; choline, 191.36 mg.
