# Supplementary material for: Subclinical doses of dietary fumonisins and deoxynivalenol cause cecal microbiota dysbiosis in broiler chickens challenged with Clostridium perfringens
Source: Front Microbiol. 2023 Apr 3;14:1106604. doi: 10.3389/fmicb.2023.1106604 (PMC10111830; doi:10.3389/fmicb.2023.1106604)
Supplement: Supplementary file 4 [file Table_2.docx]

**Supplementary Table 2. Analyzed mycotoxin content of experimental diets**

|  | **Aflatoxin**  **(ppm)** | **Fumonisin (ppm)** | **Deoxynivalenol (ppm)** | **Zearalenone (ppm)** | **Nivalenol**  **(ppm)** |
| --- | --- | --- | --- | --- | --- |
| **Starter diet** |  |  |  |  |  |
| Control | 0.04* | 0.4^1^ | 0.1 | < 0.05 | < 0.1 |
| Treatment | 0.03* | 2.8^1^ | 4.3 | 0.3 | < 0.1 |
| **Finisher diet** |  |  |  |  |  |
| Control | 0.003* | 1.5^1^ | 0.2 | 0.07 | < 0.1 |
| Treatment | 0.002* | 2.9^1^ | 4.0 | 0.4 | 0.2 |

*Total Aflatoxins (B1 + B2)

^1^ Total fumonisins (B1 + B2 + B3)

The final diets were analyzed by LC-MS/MS at Romer Labs, Union, MO, USA
